# Supplementary material for: Dengue virus infection changes Aedes aegypti oviposition olfactory preferences
Source: Sci Rep. 2018 Sep 4;8:13179. doi: 10.1038/s41598-018-31608-x (PMC6123472; doi:10.1038/s41598-018-31608-x)
Supplement: Supplementary file 1 — Supplementary Figures S1 & S2 [file 41598_2018_31608_MOESM1_ESM.pdf]

Dengue virus infection changes *Aedes aegypti* oviposition olfactory preferences

Julie Gaburro[1,2], Prasad N Paradkar[1], Melissa Klein[1], Asim Bhatti[2], Saeid Nahavandi[2], Jean-Bernard Duchemin[1, *].

Affiliations

[1] CSIRO Health and Biosecurity, Australian Animal Health Laboratory, Geelong, Australia.

[2] Institute for Intelligent Systems Research and Innovation (IISRI) – Deakin University, Waurn Ponds, Australia

Corresponding author

[*] Author for correspondence: Jean-Bernard.Duchemin@csiro.au

SUPPLEMENTARY FIGURES

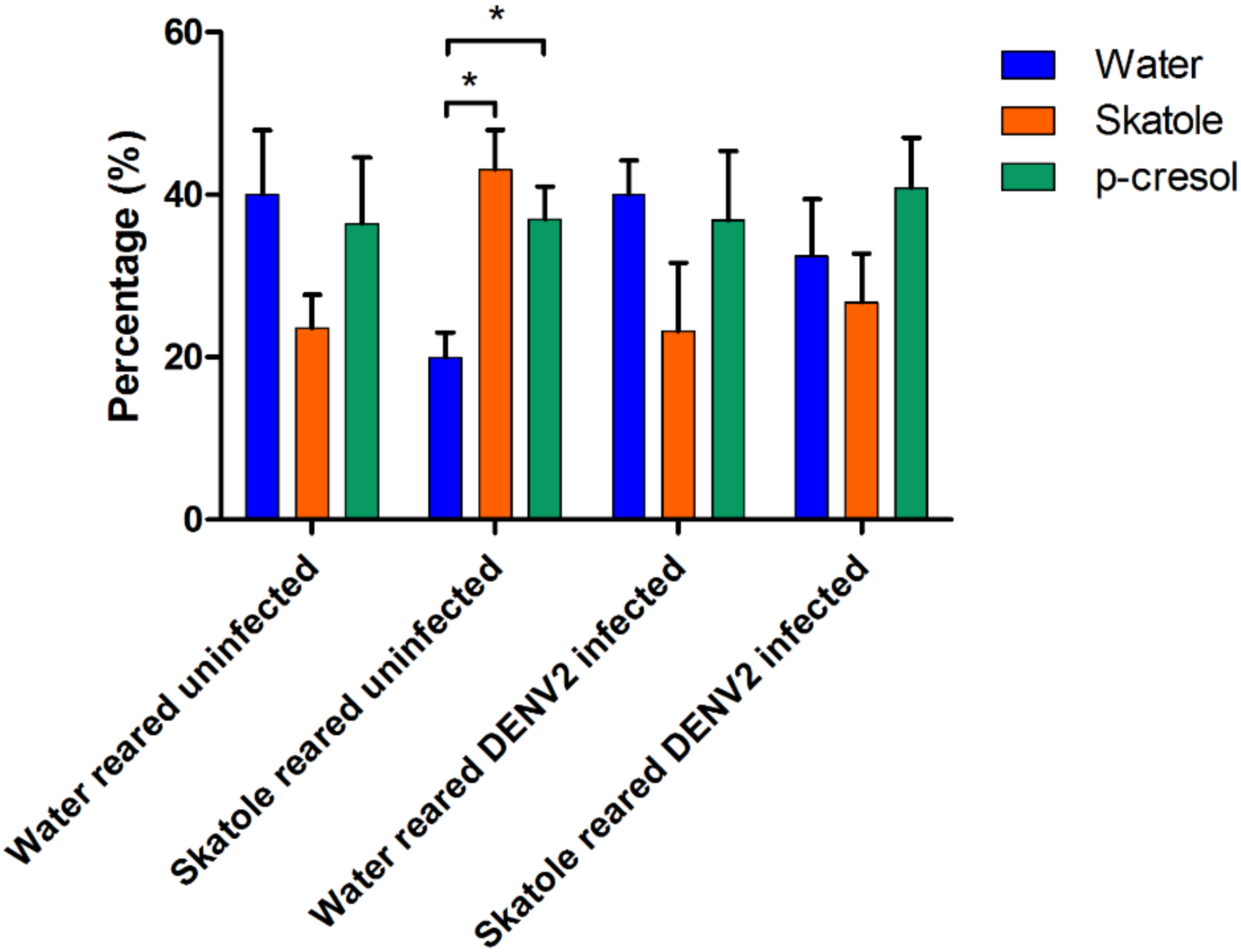

Supplementary Figure S1. Oviposition choices of infected adult *Aedes aegypti* females after a second, not infectious, blood-meal. Bar plots showing water and skatole-reared female preferences in percentage of eggs in each container after a second, not infectious, blood-meal. Data shows the mean percentage (± sem) of the four experimental replicates and *P*-values for significant differences between the egg percentages with *$P < 0.05$; **$P < 0.01$; ***$P < 0.001$.

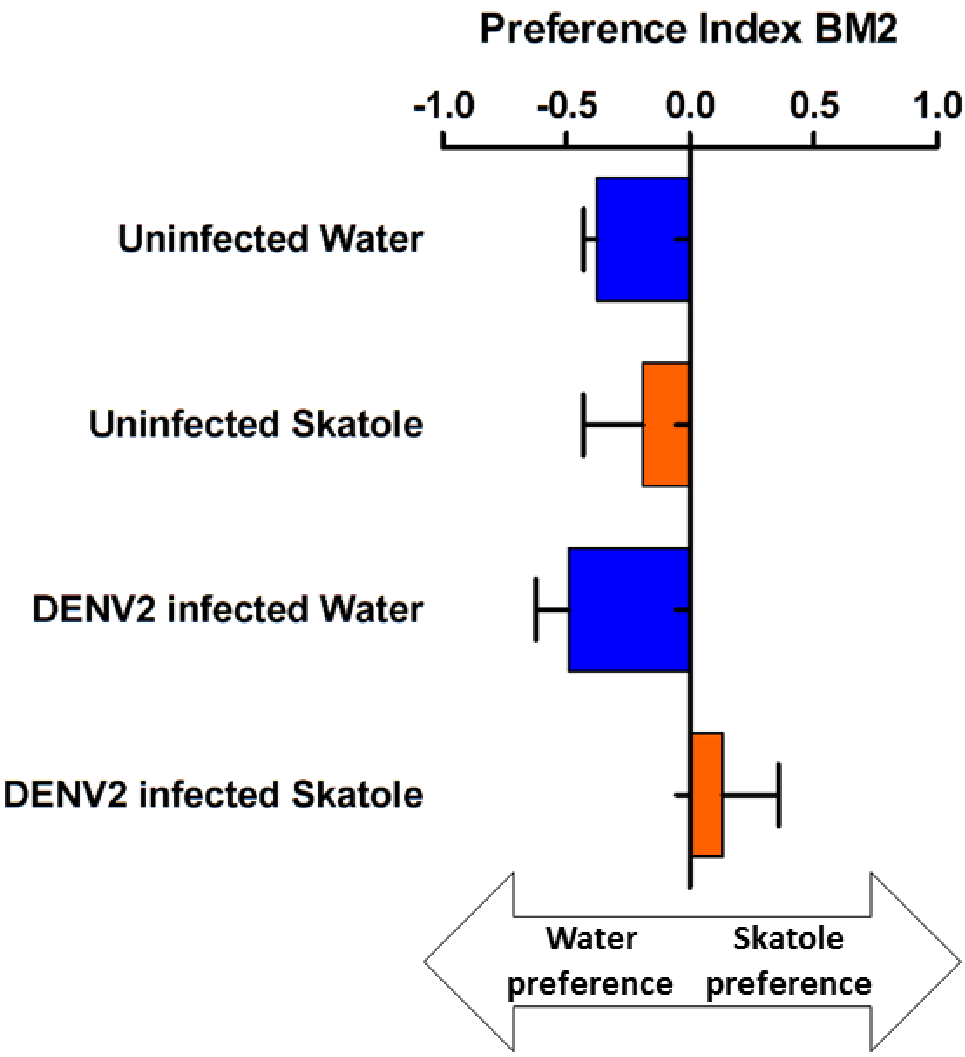

Supplementary Figure S2. Dual olfactory choices between water and skatole during oviposition period of infected *Aedes aegypti* females after a second, not infectious, blood-meal. Results of dual tests with different female groups. The Preference Index (PI) indicates females that have chosen the arm with skatole (positive PI) or water (negative PI). Bar plots indicate means of PI (± sem).
